# Iconic Athletes and the Antivaccine Movement: An Improbable Alliance That Reinforces Science Denial

**DOI:** 10.1016/j.focus.2023.100066

**Published:** 2023-01-18

**Authors:** Heslley Machado Silva

**Affiliations:** 1 Department of Science and Education, University Center of Formiga (UNIFOR/MG), Formiga City, Brazil; 2 Department of Science and Education, State University of Minas Gerais (UEMG), Minas Gerais, Brazil

In the past, the cigarette industry's marketing was strongly linked to sports, even though it was widely known that smoking worsened athletic performance and was harmful to health. Many notable sporting celebrities had their images associated with the tobacco industry, and some even died of using the products they endorsed. Fortunately, this malignant association was opposed, and since the beginning of the second decade of the 21st century, any portrayal or endorsement of addictive substances is prohibited in sporting events almost everywhere. Time, knowledge, and legislation have curbed this practice, and there has been an accompanying generalized drop in smoking rates.^1^

The coronavirus disease 2019 (COVID-19) pandemic has seen a new paradox arise, one eerily echoing those past athletes who chose to be associated with tobacco: in a crisis that should have enhanced and crystallized the value of vaccination, some athletes are instead speaking out against immunization. Just as the various vaccines were achieving significant victories against severe acute respiratory syndrome coronavirus 2 (SARS-CoV-2) in 2021 and early 2022, thereby restoring the prospect of a return to normal life by overcoming the pandemic, leading athletes in popular sports began aligning themselves with the antivaccine movement and speaking against COVID-19 vaccination.^2^

Take tennis and surfing for example. Tennis is inextricably linked with health and success, given the physical and psychological demands of the sport.^3^ Surfing is archetypically synonymous with a healthy life lived close to nature.^4^ Both sports would seem to be far from something as harmful as the antivaccine movement. Yet, in these 2 sports, 2 prominent figures emerged as symbols of a challenge to global public health during the COVID-19 pandemic.

Tennis player Novak Djokovic, poised to become one of the greatest tennis players yet, chose to take up the senseless cause of antivaccination and was literally expelled from Australia for refusing to take the COVID-19 vaccine.^5^ This image of a rebel against compulsory vaccination was immediately embraced by the advocates of supposed individual freedom to stand against immunizers. Another sportsman, Kelly Slater, arguably one of the greatest surfers to date, also allied himself with this strange phenomenon, refusing the vaccine against COVID-19, even if it meant exclusion from competitions in the sport in which he was an 11-time world champion.^6^

Endorsement of the antivaccine platform by athletes exerts a negative influence through a clumsy, flawed logic: if sports exponents, symbols of health, do not get immunized, vaccines must not be conducive to health. If that seems hard to believe, remember the athletes and the cigarettes of the fairly recent past. Images were and are worth a thousand words―often much more than rationality. They are more emotionally evocative than dry facts, however true those facts are. This flawed association particularly reverberates in a population immersed in an expanding virtual world (e.g., Generations Y, Z, and Alpha),^7^ especially the youngest of citizens. These athletes are idols of several generations, framed as opinion leaders and people to be followed, whether in lifestyle or through a social page, each with millions of virtual followers.^8^

We cannot attribute the growth of the antivaccine movement or the low demand detected worldwide for immunization entirely to athletes. It might have been expected that after the evident victories in global public health provided by the vaccination campaigns against SARS-CoV-2 in the years 2020 and 2021,^9^ vaccines would be trusted, but what we see instead is that the forces that oppose immunization have attained mass credibility using new forms of communication.^10^ Their ideological position, based on supposed individual freedom,^11^ now has athletic supporters who exert great social influence through the channels of social media.

The return of measles^12^ does not seem to scare as it should, and the failure of the proposed drugs against COVID-19^13^ seems to have been ignored. An unwelcome return of the scourge of polio^14^ causes no great disquiet. Science denialism is communicating well and mounting unexpected new arguments. It used to be possible to fund sports to obtain an indirect advertisement for smoking; fortunately, this has ended, and today, in most countries, tobacco use disorder is declining. The antivaccine movement pays nothing to garner support across social networks and the wider Internet, combining false arguments with genuine news to create a superficially plausible but deeply toxic amalgam that feels like sense to a proportion of the global population. Conspiracy theories arise and multiply in this context and further reinforce false ideas about vaccines.

How should we deal with this new challenge? Multiple approaches will be needed―one that is perhaps not new^15^ but rather has a new dimension. Massive and ingenious campaigns will be needed to enlighten the population, but the traditional media are losing ground: few people watch television, and even fewer read newspapers and articles, while social networks seem omnipresent and omnipotent.^16^ It is important to seek new and exciting ways for the scientific field to be able to insert itself into these media to promote persuasive public health policies and for vaccination campaigns to find ways to communicate through now ubiquitous social networks. It will be necessary to convince people that this is not a matter of freedom (either for outstanding athletes or any other citizens) but rather one of individual and collective civic duty, because if polio returns or a new variant of SARS-CoV-2 appears, the whole of society will be penalized, including those same sportspeople, along with everyone else, and all their affected families. We must promote once again the victories that vaccination has provided to the world.^17^ Otherwise, this almost medieval movement will continue its march, leading us inexorably to scapegoating and obscurantism in a world wracked by recurrent plagues.

Academic journals should be concerned with reminding the scientific, public health, and medical communities about their collective responsibility to counter the misinformation that denialism fosters and propagates in the general population and even within their own ranks. If it is thought wrong for athletes to align themselves with the antivaccine movement, what can we say when parts of the medical community, in Brazil for example, does likewise? During the pandemic, many of these doctors efficiently deployed social networks and the Internet to spread false news about vaccine safety and repeated debunked theories about drugs proven to be ineffective against COVID-19.^18^ This phenomenon was not exclusive to Brazil: it is happening in various parts of the world, amounting to an infodemic^19^ in the health arena. It will take a collective effort to assure that doctors and all health professionals follow the science and not be spreaders of fraudulent news. These professionals’ prestige makes them particularly socially credible, and therefore, initial and continuing training to protect them from widespread misinformation may be indicated. This, in turn, may also help protect the societies in which these health professionals live and work from the consequences of vaccine misinformation.

## References

[1] WHO, WHO global report on trends in prevalence of tobacco smoking 2000–2025, Second edition, 2018, World Health Organization; Geneva, Switzerland https://apps.who.int/iris/bitstream/handle/10665/272694/9789241514170-eng.pdf?ua=1, Accessed November 15, 2022.

[2] Beltrão RPL, Mouta AAN, Silva NS (2020). Perigo do movimento antivacina: análise epidemio-literária do movimento antivacinação no Brasil. Rev Eletrônica Acervo Saúde.

[3] Elliott B (2006). Biomechanics and tennis. Br J Sports Med.

[4] Britton E, Foley R. (2021). Sensing water: uncovering health and well-being in the sea and surf. J Sports Soc Issues.

[5] Burki TK. (2022). Vaccination in the world's top athletes. Lancet Respir Med.

[6] Boyd D. (2009).

[7] McCrindle M, Wolfinger E. The ABC of XYZ: Understanding the Global Generations, 2009, McCrindle Research; Norwest, Australia. ISBN: 978 1 74223 023.

[8] Goodyear VA, Armour KM. (2019). http://library.oapen.org/handle/20.500.12657/28205.

[9] Mathieu E, Ritchie H, Ortiz-Ospina E (2021). A global database of COVID-19 vaccinations [published correction appears in *Nat Hum Behav*. 2021;5(7):956–959]. Nat Hum Behav.

[10] Burki T. (2020). The online anti-vaccine movement in the age of COVID-19. Lancet Digit Health.

[11] Wolfe RM, Sharp LK. (2002). Anti-vaccinationists past and present. BMJ.

[12] Portnoy A, Jit M, Ferrari M, Hanson M, Brenzel L, Verguet S. (2019). Estimates of case-fatality ratios of measles in low-income and middle-income countries: a systematic review and modelling analysis. Lancet Glob Health.

[13] Hallal PC, Victora CG. (2021). Overcoming Brazil's monumental COVID-19 failure: an urgent call to action. Nat Med.

[14] Thornton J. (2019). Polio returns to the Philippines. Lancet.

[15] Silva HM. (2021). 100 years later, little has changed in Brazil: disinformation and pandemic. Afr Health Sci.

[16] Brumfiel G. (2009). Science journalism: Supplanting the old media? [published correction appears in *Nature*. 2009;458(7238):562]. Nature.

[17] Silva HM. (2021). The historic success of vaccination and the global challenge posed by inaccurate knowledge in social networks. Patient Educ Couns.

[18] Silva HM. (2022). Antibiotics against viruses: Brazilian doctors adrift. Infect Control Hosp Epidemiol.

[19] Orso D, Federici N, Copetti R, Vetrugno L, Bove T. (2020). Infodemic and the spread of fake news in the COVID-19-era. Eur J Emerg Med.

